# Improving How Healthcare Staff Support the Psychological Well‐Being of People Living With Post‐Stroke Aphasia: A Co‐Created Study

**DOI:** 10.1111/hex.70303

**Published:** 2025-05-28

**Authors:** Sarah Northcott, Amanda Comer, Abi Roper, Lydia Davis, Katerina Hilari

**Affiliations:** ^1^ City St George's, University of London London UK

**Keywords:** aphasia, co‐production, emotional recovery, humanising care, psychological well‐being

## Abstract

**Background and Aims:**

Aphasia, a language disability, is common following a stroke. People with aphasia are at risk of becoming depressed and isolated, yet due to their communication difficulties, healthcare staff find it challenging to support their emotional well‐being. This study aimed to explore what people with aphasia and their families consider important when training healthcare staff to support their psychological well‐being post stroke.

**Methods:**

We ran co‐design workshops with six stakeholders with lived experience: four people with aphasia and two family members. The content of the workshops was allowed to evolve in a collaborative manner, with an assumed equality between the facilitators and lived experience stakeholders. Workshop material was analysed using Framework Analysis. We then co‐produced four films to raise awareness and train healthcare professionals.

**Results:**

Five main themes from the workshops were: (1) interactions with healthcare staff that support psychological well‐being (e.g., listening with empathy, seeing patients as people, hope and encouragement, kindness and knowledge of aphasia); (2) interactions with healthcare staff that damage psychological well‐being (e.g., feeling told off, being talked about and not included, not feeling listened to, not being supported to communicate and not feeling treated like a human being); (3) experiences of psychological therapy and mental health services; (4) who should provide psychological support and (5) influencing healthcare practice. The four films emphasised the personal journeys of lived experience stakeholders and their accounts of interacting with healthcare staff.

**Discussion:**

Lived experience stakeholders felt strongly that their messages should be heard by all healthcare staff, not just those who elect to go on specialist training courses. They considered that supporting emotional well‐being is the responsibility of all staff within stroke care.

**Patient or Public Contribution:**

People with aphasia and family member stakeholders shaped all aspects of this study; outputs were allowed to evolve in response to their priorities. Initially, the researchers had anticipated that the focus would be on specialist training courses in psychological therapy; this shifted to a new focus on influencing how all healthcare staff interact with patients, including both non‐clinical staff and staff who would not elect to go on a specialist training course. The co‐produced films were a direct result of lived experience stakeholders' suggestions and priorities.

## 1 Introduction

Aphasia is a language disability that can affect speaking, understanding, reading or writing and is common post stroke: median frequency of post‐stroke aphasia has been estimated at 30% in acute settings and 34% in rehabilitation settings [1]. Having aphasia makes someone vulnerable when receiving healthcare, as healthcare staff find it difficult to communicate successfully with them [2]. This is exacerbated when the intervention is language‐based, such as with most psychological therapy and mental health interventions [3]. As such, people with aphasia often do not have equitable access to psychological support during their recovery post stroke [4, 5]. This study aimed to work with people with aphasia and their families on what they considered important when training healthcare professionals to support emotional recovery post stroke and aphasia.

Having a stroke is a risk factor for depression: prevalence of depression is estimated to be 29%, a figure that remains stable up to 10 years after a stroke [6]. People with aphasia appear to be particularly at risk of depression, with rates of 43%–70% [7]. They are also at high risk of anxiety, with 44% of people with aphasia found to have significant anxiety [8], and having a reduced social network [9]. Not only is it an isolating and distressing condition, but it also makes accessing mental health services more challenging. Mental health professionals describe finding it difficult to adapt their work for people with aphasia [3]; further, people with aphasia are less likely to be referred due to the language disability [4]. Speech and language therapists are the key professionals within the stroke team who support communication, yet describe various barriers to addressing psychological needs themselves, including a lack of confidence, inadequate specialist training and a lack of time and institutional support [5, 10, 11]. They also describe their clients' challenges in accessing appropriate mental health support [5, 10, 11].

Research suggests people with aphasia would value access to a ‘stepped care’ model of psychological support [12]. In the stepped care model, when someone has severe, persistent distress, including suicidal ideation, they are triaged to specialist mental health services (psychiatrist and stroke‐specialist psychologist); for those with moderate‐level needs, including symptoms of depression that compromise rehabilitation, it is recommended they see either a clinical psychologist or other stroke healthcare professional who has specialist training and support from a psychologist. For common mild, or ‘sub‐threshold’, mood problems, it is suggested that the whole stroke team is responsible for their psychological care, for example, through active listening, problem‐solving, providing information and facilitating peer support [13].

There has been encouraging research that suggests it is possible to adapt psychological therapies so that they work well with people with aphasia [14] and that with appropriate training speech and language therapists can deliver psychological therapy, so long as they have access to real‐time support and specialist supervision from a mental health professional [15, 16]. There has also been encouraging research on the feasibility of training SLTs in counselling skills [17]. Given that speech and language therapists currently lack confidence in psychological therapy [18] and that mental health professionals find it challenging to adapt their work for people with aphasia [3], there is a clear need to upskill the workforce in a sustainable way. Part of the solution is likely to be the provision of training, yet it is not known what people with aphasia and their families consider important to include in training. For training to target outcomes that are meaningful to end users, we worked collaboratively with people with aphasia and their families as active and equal partners, foregrounding their experiences and values within the research process from an early stage. Taking a co‐production approach can produce research outputs that are more relevant, acceptable, usable and impactful [19].

### 1.1 Aims

The original aim was to explore the views of people living with aphasia and family members on what they wanted included within specialist training for healthcare professionals who elect to go on advanced courses in brief psychological therapy approaches, such as solution‐focused brief therapy. However, through the collaborative process, the focus shifted to training or influencing healthcare staff, both clinical and non‐clinical, who would *not* elect to attend a specialist training course. A further aim that evolved through the workshops was to co‐produce videos highlighting interactions within healthcare which damage or support emotional recovery.

## 2 Materials and Methods

### 2.1 Study Overview

Four online co‐design workshops were held with lived experience stakeholders, facilitated by two researchers. They took place over a 6‐week period. An output from these workshops was that lived experience stakeholders wanted to make videos to communicate key messages to influence healthcare practice, leading to four co‐produced films. The final stage of the research process was agreeing on the dissemination strategy.

### 2.2 Stakeholders

There were three stakeholder groups: stakeholders who had lived experience of aphasia, researcher stakeholders and video production stakeholders.

Lived experience stakeholders: Everyone approached by the research team wanted to take part in the workshops. They were all known to the research team through previous research activity at the university. The research team were mindful of including people from diverse backgrounds. As a result, stakeholders were from different ethnic backgrounds (white and Asian), sex and age (working age and over 65); living situation (living alone and living with family); and geographical location (urban and rural). There were four people with aphasia and two related family members. All members with aphasia had experience of being involved in previous research projects as participants. Two people with aphasia and one family member had experience of taking on advisory roles within research; for the other three members, this was their first experience in an advisory, collaborative role. All stakeholders were able to access online meetings and attend all four workshops. Five workshop members subsequently volunteered to contribute to the video co‐production: four people with aphasia and one family member.

Researcher stakeholders: The five researchers were all female speech and language therapists with extensive experience of working with people with aphasia. They included early career, mid‐career and senior researchers and brought expertise in qualitative and co‐design methodology. Two researcher stakeholders, S.N. and K.H., had previously led research projects investigating psychological interventions for people with aphasia. S.N. and L.D. facilitated the workshops; S.N. and A.C. facilitated the video production process.

The video production company, Copperwheat Media, became an additional stakeholder, bringing relevant creative experience and also lived experience from their previous careers within healthcare (paediatric oncology nurse and children's mental health worker). They shaped video content, for example, the decision to foreground stories rather than didactic instruction.

### 2.3 Workshop Process

The workshop process was iterative: after each workshop, the two facilitators reflected on the themes to emerge, which were then checked and refined in subsequent workshops. The content of the workshops was allowed to evolve in a collaborative manner. Table 1 details the initial plan for the workshops and how this changed in response to the priorities of the lived experience stakeholders. The International Association for Public Participation's (IAP2) Public Participation Spectrum [20] suggests there are five levels of engagement: inform, consult, involve, collaborate and empower. We initially conceptualised the study as collaborative (partnering with end users in each aspect of decision‐making). However, given that lived experience stakeholders suggested and determined the final outputs, the level of engagement arguably evolved to become a model of empowerment (overall shape of project decided by end users).

**Table 1:** Workshop content.

Workshop	Initial plan	Changed plan (modified to reflect priorities of lived experience stakeholders)
Workshop 1	Introductions, group preferences Topic 1: Emotional support from healthcare professionals Topic 2: What helps/doesn't help	Introductions, group preferences Topic 1: Sharing stroke and recovery stories; sharing personal information Topic 2: What healthcare staff do that supports emotional recovery post stroke Topic 3: Negative experiences in the hospital and with mental health professionals Topic 4: Who to target with training
Workshop 2	Topic 1: Who should provide emotional support Topic 2: What is important in a healthcare professional	Topic 1: Negative and positive interactions with healthcare staff Topic 2: Experiences of aphasia, and how this affects interactions with healthcare staff Topic 3: Prognosis, recovery, loss and hope Topic 4: Who should provide emotional support both in the hospital and back home Topic 5: Important qualities in healthcare staff
Workshop 3	Topic 1: views on providing solution‐focused brief therapy training to speech and language therapists and other healthcare professionals Topic 2: What is important to include in training; ideas for training materials	Topic 1: Views on ways to influence healthcare practice Topic 2: Priorities on whose practice to influence Topic 3: Planning videos; purpose of videos Topic 4: Mental health services and aphasia
Workshop 4	Topic 1: Member checking Topic 2: Views and priorities for funding applications	Topic 1: Revisiting and refining themes from previous workshops Topic 2: Views on how to share key messages; who to target Topic 3: Views and priorities for funding applications

There were four online workshops: the decision to have them online facilitated more inclusive access (e.g., those with poor mobility and geographical diversity). Each workshop was approximately 1 h long: the length of meetings was decided by consensus with the lived experience stakeholders. In addition to the workshops, one person with aphasia also emailed S.N. with further reflections. Led by the individual needs and preferences of stakeholders, S.N. met individually with some to offer further support, for example, if they had shared something personal in a workshop. How the group functioned was determined collaboratively, for example, one stakeholder with aphasia explained that it was immaterial whether someone was a company executive or a refuse collector—everyone mattered. Others noted the importance of not feeling judged, of feeling included and supported, and smiling at each other. During the workshops, lived experience stakeholders encouraged, empathised and complimented each other, celebrating each other's achievements. They shared both painful and positive experiences relating to the stroke and life more generally (e.g., taking up running, their role in a street party and their local football team). This is in line with foundational principles of stakeholder engagement in research on respect, equitable power balance and trust [21].

Workshops were not recorded; however, detailed notes were made by the co‐facilitator, L.D. These notes were used to identify themes, plan workshops and subsequently plan the videos. Brief aphasia‐accessible agendas with key topics were emailed before each workshop, and an aphasia‐accessible summary was shared after each workshop. Members requested this to aid further reflection between workshops. Other steps were taken to facilitate people with aphasia: the lead facilitator, S.N., adjusted her own language, checked her understanding of others' points, wrote key words and topics on PowerPoint slides supported by pictorial images, made sure people had time and space to contribute, and actively valued different perspectives. One person with aphasia emailed S.N. before and after meetings, and on occasion, requested S.N. to summarise her emails for the group to discuss.

### 2.4 Analysis

**Figure 1:**
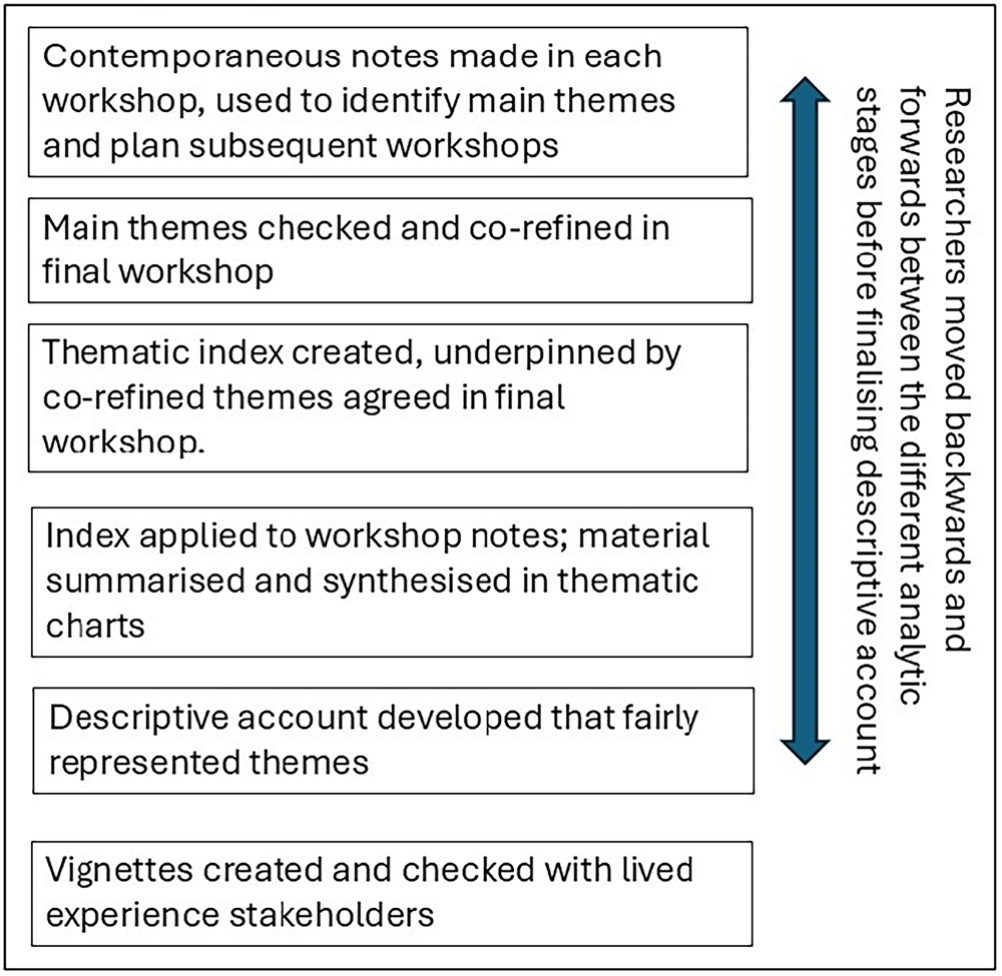
Analytic process.

Detailed contemporaneous notes were made during the workshops. After the third workshop, the facilitator, S.N., analysed the material for key messages. These were presented to lived experience stakeholders in the final workshop for further refinement. Subsequently, a researcher not involved in the workshops (A.C.) independently analysed the notes from all four workshops and also contemporaneous email correspondence, using Framework Analysis [22]. This is a systematic and inductive approach for producing descriptive accounts of qualitative material widely used within healthcare research [23]. Initially, A.C. familiarised herself with the contemporaneous workshop notes, creating a draft thematic index. This was discussed and refined with S.N. before it was used to index the workshop material, such that each phrase was labelled within the thematic index. Thematic charts were constructed whereby chart headings matched the thematic index, with each workshop member allocated a row, and their material synthesised and placed in the relevant chart. This matrix‐based method of organising the data enabled the researchers to explore the range and patterns of views. Given that meaning was built and refined through the workshop process, the researchers moved backwards and forwards from the contemporaneous workshop notes, the within‐workshop member checking process, and the higher‐order themes to ensure the final descriptive account was a fair reflection (see Figure 1). To reduce bias, this iterative process was conducted with reflective discussion between A.C. and S.N.

### 2.5 Video Production Process

The decision to make films came through the co‐design process: workshop members intended the films would illuminate the core messages that they considered important for healthcare staff to hear (for example, positive and negative interactions within healthcare). The content further evolved through the film co‐production process. All stakeholders (researcher, lived experience and video production) were involved in planning and deciding on content for the videos through online meetings and emails. The researchers worked closely with Copperwheat Media to discuss how lived experience stakeholders would be supported through the process and shared ideas on how to facilitate their communication. All stakeholders were involved in the editing decisions that led to the final videos and agreed on a dissemination strategy, including the use of social media and publication.

#### 2.5.1 Ethical Considerations

Workshop members were not considered research participants, but rather expert advisors. We conceptualised our approach to collaborative working with key stakeholders as patient and public engagement rather than primary research. In line with guidance from the Health Research Authority (www.hra.nhs.uk), we therefore did not apply for ethical approval and did not formally consent workshop members into the project as ‘participants’. We did, however, talk through the nature of the project carefully with all lived experience stakeholders and what to expect to ensure they were fully informed in their decision to become involved. They were aware that they could change their mind and did not need to attend all the workshops. In recognition of their role, they were paid £25 an hour. Workshops were not recorded to minimise the data collected, although stakeholders gave consent for L.D. to take notes. Personal information was not collected.

For the video production, we followed City St George's protocol for gaining informed consent when filming members of the public, which we adapted to be accessible for people with aphasia. A member of the research team (S.N. or A.C.) met individually with each lived experience stakeholder to talk over what would be involved and gain informed written consent and was present during filming to provide support. The researchers checked stakeholder preferences: ‘talk to camera’, which would mean waiving their anonymity or having their story spoken by someone else, preserving anonymity; whether and how they wanted their name acknowledged at the end of the film. Their preferences were checked and rechecked at multiple points: before filming, on the day of filming, during the editing process, before dissemination (including written publications). They were reassured they could change their mind and request some or all of their footage removed. Before making the films public, the researchers took care to reconfirm consent; researchers reiterated that once the films were released, it would not be possible to reverse their decision. The researchers aimed to ensure all lived experience stakeholders felt in control throughout, had agency and ownership of the process, and could take pride in the final product.

## 3 Results

### 3.1 Descriptive Account of Co‐Design Workshops

There were five main themes from the workshops: interactions with healthcare staff that supported or damaged well‐being, experiences of mental health support, who should provide psychological support, and influencing healthcare practice. As the workshops were not recorded, verbatim quotes were not used. Instead, themes are illustrated through vignettes (see Table 2) The vignettes highlight material that stakeholders prioritised for inclusion in the films; vignette wording was agreed with stakeholders. The views of the lived experience stakeholders only are reported below.

**Table 2:** Illustrative vignettes for Themes 1 and 2.

Vignettes (material taken from workshops and video production; wording agreed with stakeholders)	
John (supports Nottingham Forest football club; dug a pond and laid decking one‐handed)	I had my stroke on Christmas Day. The nurses was good, yeah, always good morning, or good day, they was chatty, chatty with me, lots of jokes. I didn't answer because I couldn't speak, but it was a good feeling. The doctors, they came to me, a doctor and junior doctors. They talk about me. They left my room. Not how are you? They should greet you, smile, say ‘good bye’.
Mei‐Lin* (worked as an interpreter before her stroke, speaks five languages, loves Beethoven) *pseudonym	After my stroke, I can't speak or move, but I had my emotions. I was lying there, the doctor said to his students, ‘see this person’. I can hear, I can understand. The doctors and nurses didn't treat me as a human being, I was treated as a piece of meat. The only person who is nice to me in the hospital is a kitchen helper. He delivered the food. One day, he spoke to himself in Spanish. I heard, and I laughed. He is so excited. He began to speak in Spanish to me. We shared our emotions. A kitchen helper, so humble yet with a big heart. He brings me the thing I need: having a heart, for everybody.
Jo Doody (daughter was 8 when she had her stroke, makes pottery and upcycles furniture one‐handed)	I was in the hospital for 6 months, it was touch and go. When I was in hospital, I couldn't do anything, I was very poorly. There was a nurse. She washed me, dressed me—she was rough. I was embarrassed. I couldn't speak, I couldn't tell anyone. Another nurse, she was gentle, so nice, she did my make‐up, washed my hair, she was amazing. In the beginning I was crying so much, there was a doctor, she was so kind, so patient.
Michael (volunteered at Citizen's Advice Bureau; runs regularly, including in park run)	My speech therapy, it was like speech therapy and therapy because I could talk to her about problems in the real world. If I had problems with anger or frustration, I could tell her about them. She was really good at listening. After my stroke, I kept a diary. Two months after my stroke I've written: ‘98% of recovery occurs within 3 months.’ I don't remember who said it to me. Then I've written: ‘I only have one month.’ I was only in the low percentages. What I wanted was someone to tell me, you're doing well, you're making good progress.
Paula (family member; works as a healthcare data analyst)	When John was in the hospital, I chatted to the newly qualified physiotherapist, we used to have a giggle together while John was on the treadmill, just normal stuff. It helped me to cope with the life I'd given up. When she left her post, I burst into tears: I think she was surprised, she didn't realise how much those chats had helped me. The assisted discharge team were also very nurturing. always asked, and how are *you* doing, in a way that gave me permission to say, actually, I'm not doing OK. When a stroke happens to you for the first time, you don't know the rules, you don't know what's expected. I think staff sometimes forget that. There was a time when John was really upset, I was sitting on his bed, trying to comfort him. A nurse walked past, put their head round the door and said ‘oi, get off the bed’, and walked off. No explanation, no checking if I was OK, just abruptly told off as if I was a child.

#### 3.1.1 Theme 1: Interactions With Healthcare Staff That Supported Emotional Well‐Being

*Listening with empathy*. Stakeholders agreed there was value in feeling understood and that healthcare staff were really listening. When a member of staff in a healthcare setting was perceived as sympathetic, it was considered therapeutic to express difficult emotions such as frustration, anger and distress, to cry and feel supported and reassured; and it was appreciated when counselling had been integrated within rehabilitation therapy sessions. Stakeholders suggested staff ask both the person with aphasia and also family members ‘and how are you?’, in a way that enables the person to share how they wre really feeling.

*Making an effort to enter someone's world, seeing patients as people*. Stakeholders suggested it supported well‐being when healthcare staff took an interest in their patients, for example, who they were before the stroke, their interests and what matters to them. It was considered valuable when staff talked with patients as people, with whom they could laugh and chat, and both the staff member and the patient shared something about themselves.

*Being given hope and encouragement; meaningful therapy goals*. Enabling patients to notice progress, working towards therapy goals that mattered to the patient and supporting patients to have hope for the future were all perceived as helpful.

*Kindness; feeling valued and respected*. Stakeholders agreed that it supported well‐being when healthcare staff had a warm, gentle, and friendly manner and smiled. Listening and respecting patient views, for example, around future care preferences, along with small acts of kindness, were perceived to have a positive impact.

*Knowledge of aphasia*. Understanding aphasia enabled healthcare staff to support patients in expressing opinions, for example, on hospital transfer options. Stakeholders considered that it had a positive impact when staff gave people with aphasia time, had skills in enabling people with aphasia to communicate and understood that people with aphasia were still intelligent people with feelings and thoughts.

#### 3.1.2 Theme 2: Interactions With Healthcare Staff That Damaged Emotional Well‐Being

*Feeling told off*. Hospitals were perceived to have multiple rules. Stakeholders agreed it was distressing and infantilising to be reprimanded or told off for unknowingly breaking a rule.

*Being talked about and not included*. It was dehumanising to be talked about but not included or even acknowledged, for example, on ward rounds.

*Poorly handled conversations around prognosis and future recovery*. Misleading and sometimes highly specific predictions for future recovery could damage well‐being. Stakeholders shared that being told they would make a near full recovery could lead them to feel like a failure when they did not; conversely being told they would never be able to achieve something again, such as driving, or that they could expect no further recovery beyond a certain time window, mostly turned out to be untrue but at the time had a damaging impact on morale.

*Not feeling listened to; arrogant or impatient manner*. Stakeholders shared that it had been distressing when their views and experiences had been dismissed or when healthcare professionals had an arrogant manner when discussing their care.

*Not being supported to communicate*. When healthcare staff lacked knowledge of aphasia, it compromised the person with aphasia's ability to engage with them, express care preferences or even meal‐time preferences: this had a detrimental impact on psychological well‐being.

*Rough manner; not feeling treated like a human being*. Receiving personal and medical care from healthcare staff who had a rough or uncaring manner was deeply distressing. The aphasia appeared to exacerbate this, as it could make it hard to challenge the behaviour or explain to anyone what had happened. At its worst, it could make a person feel like a ‘piece of meat’, defenceless, no longer treated as a human with feelings.

#### 3.1.3 Theme 3: Experiences of Psychological Therapy and Mental Health Services for People With Aphasia and Their Families

Brief psychological therapy or counselling was experienced as helpful when it came at the right time, the therapist understood about stroke and aphasia and listened and gave space for the person to explore how to live with the stroke. Stakeholders considered it important that mental health professionals listened to their clients' preferences and respected their narratives. One stakeholder shared how she had experienced mental health services as damaging. She felt monitored and lectured, without her own expertise and personhood being validated.

#### 3.1.4 Theme 4: Who Should Provide Emotional Support and When Following a Stroke

Stakeholders considered that everyone within healthcare could have a role in supporting emotional well‐being post stroke, both within hospital and community services. While this included mental health and other healthcare professionals, everyone the person with aphasia and family encountered had the potential to positively impact well‐being, for example, a kind word from the person bringing the tea trolley. Conversely, post stroke was a vulnerable time for people, and all healthcare staff also had the capacity to damage psychological well‐being. Others perceived to have a role in supporting emotional recovery were family, friends and peer support from others living with aphasia.

In terms of the timing of emotional support, humanising healthcare services was considered important throughout the stroke pathway. Coming home from the hospital and being discharged from stroke rehabilitation were considered particularly difficult moments. Access to psychological services that understood aphasia was considered problematic, and there was a perceived need for specialist psychological support to be accessible when needed, including in the chronic stage.

#### 3.1.5 Theme 5: Influencing Healthcare Practice

While there was support for delivering specialist psychological training to speech and language therapists, the priority for workshop members was reaching those with poor understanding and awareness whose actions had the potential to damage psychological well‐being and influencing the culture of healthcare more broadly. Potential strategies included creating a brief video to be included in hospital mandatory training, creating leaflets, using social media and working with hospital appraisal systems. A video with personal testimony was considered a powerful tool to influence healthcare practice, versions of which could potentially be used within training and raising awareness more generally.

### 3.2 Narrative Summary of Co‐Produced Films

Four films were created with stakeholders with lived experience (see Table 3). Three people with aphasia and one family member chose to speak to the camera, inviting the film company into their homes. One person with aphasia requested that the researcher (S.N.) share her story on her behalf, agreeing the wording with the researcher.

**Table 3:** Film content.

Format: humanising film work that centred the person and their life.
Location: people's homes; one video (18‐min training video) also included a researcher, S.N., talking in a university setting.
Video 1: ‘Psychological journeys after stroke and aphasia: Jo and Michael's stories’ Intimate storytelling video outlining Jo and Michael's initial stroke experiences, interactions which supported/hindered their psychological well‐being, and finding ways to live well after their stroke. 7 min https://bit.ly/aphasiajourney
Video 2: ‘A family member's perspective on humanising stroke care’ Foregrounds a family member's experiences, including: communicating with medical staff; the impact of feeling told off; the value of asking family members how they are; the strain on family members and holding on to hope. 4 min https://bit.ly/aphasiafamily
Video 3: ‘Psychological care after a stroke and aphasia: what we can all do to help’ A training video aimed at healthcare staff and students. It covers background information about aphasia, what healthcare staff can do to support emotional well‐being, what healthcare staff do that damages psychological recovery and three steps staff can take tomorrow to support patient well‐being. 18 min https://bit.ly/aphasiacare
Video 4: ‘A Christmas Day stroke: John's story’ John's life before the stroke, the impact of the stroke and his life now. 4 min https://bit.ly/aphasiaOK

The films took an intimate storytelling approach that emphasised the personal journeys of the stakeholders, woven through with their experiences of interacting with healthcare staff and psychological recovery. The films were shot in people's homes and included footage of them engaging in valued activities such as gardening or running in local streets. This approach emphasised two of the key themes from the workshops: the importance of noticing and valuing the person and what matters to them; and the role of hope for a life worth living after the stroke. Table 3 provides a summary of the content of the four films.

## 4 Discussion

People with post‐stroke aphasia are at risk of psychological distress, exacerbated by poor interactions with healthcare staff. This study worked collaboratively with people with aphasia and family members to explore their views and priorities for training and influencing healthcare staff to better support psychological well‐being post stroke. Workshops identified ways in which healthcare staff support emotional recovery, such as listening to the ups and downs, noticing and valuing their patients as people, and supporting the person with aphasia to communicate. They also identified ways in which healthcare staff damage psychological well‐being, such as: telling people off; not acknowledging or including people with aphasia in conversations; and a rough, impatient or uncaring manner. The priority of workshop members was to humanise stroke care and challenge psychologically damaging behaviours. This led to co‐producing a series of four films to be used in training and raising awareness.

A key theme from this study was that negative interactions with healthcare staff could have a detrimental impact on well‐being, both for the person with aphasia and their family member. A review of qualitative studies exploring stroke survivors' experiences of rehabilitation similarly described the strong negative impact on mood and motivation when staff had authoritarian attitudes and decision‐making processes, dismissing patient's goals and autonomy [24]; carers also describe the negative psychological impact of demeaning, adversarial or disparaging interactions with stroke staff [25]. After a stroke, people describe feeling shock, confusion and panic, exacerbated by being in the unfamiliar alien environment of the stroke unit [12, 26]. Given their need for reassurance [26], if they instead feel ignored or treated as less than human, it follows that they will be vulnerable to worsening distress.

Conversely, the study underlined the impact of positive interactions with staff and how seemingly mundane conversations that conveyed that staff were interested in them as a person, as well as encouragement, warmth and empathy, could make a difference. Other research has also found the positive impact of stroke staff valuing relational work and taking a holistic interest in their patients [27]; how being treated with friendliness and kindness and being responded to as an individual on a stroke unit helped with emotional adjustment [28]; and that receiving reassurance and feeling that they were not on their own supported stroke patients with the strong emotions they were experiencing [26].

Consistently, research suggests that healthcare staff want to support emotional well‐being and see this as part of their role [5, 18, 29]. When they are unable to do so, it can lead to feelings of guilt, inadequacy and moral injury [29]. Despite this desire to support well‐being, there are factors that militate against this happening within stroke care. Staff describe pressure to ‘rush patients through’ to meet targets, making it challenging to build relationships [26]. There is a perceived lack of time for relational care and the need to protect time to complete discipline‐specific tasks [2, 26, 29], with limited, scripted conversations that focus on physical function [30]. More generally, there is a focus on physical care within stroke units: mood is not always seen as a high priority by the wider stroke team, nor as necessarily within the remit of people's roles [4, 29]. Performance indicators and discharge criteria focus on biomedical and impairment‐based frames of reference, driving treatment priorities [29, 31]. Even where staff consider it important, there is a sense that time spent supporting patient well‐being is ‘unsupported and invisible’, since it is not easy to demonstrate, nor is it recorded in patient records or key performance indicators [29]. Further factors may be a risk‐averse culture that can be in conflict with patient autonomy [24] and an unstimulating environment [24, 26, 31]. Finally, healthcare staff describe feeling that they lack training, knowledge, confidence and skills to provide emotional support [4, 5, 29].

An additional barrier to addressing emotional well‐being is likely specific to the aphasia. There is evidence that stroke staff lack confidence and skills in communicating with someone with aphasia [2]. Stroke staff describe conversations with people with aphasia as time‐consuming and challenging and use strategies to avoid or limit communicating with people with aphasia. These include avoiding unplanned conversations, focusing conversations on time‐bound needs‐based or discipline‐specific topics, avoiding open‐ended or complex topics, and avoiding engaging in normal social interactions [2]. While feeling bored, alone and distressed on a stroke unit is a frequently described experience [24], having aphasia appears to worsen this, such that they have been described as the ‘forgotten patients’ [30].

This early period after the stroke represents a psychologically vulnerable time for both the person with aphasia and family members, who are often feeling overwhelmed, frightened and anxious [12, 24, 25, 26]. The lived experience stakeholders felt strongly that in this context, it matters that care is provided in an emotionally sensitive manner. Since the 2013 Francis report [32] identified serious deficiencies in relational aspects of care within the UK National Health Service (NHS), there has been a drive within the United Kingdom to improve compassionate care. This has been mirrored globally with a movement towards person‐centred and humanising care, where the values and views of the patient and family are foregrounded in clinical care and decision‐making [33, 34]. The humanising care theoretical framework has been developed to guide health practitioners on what enables a person to feel human within healthcare, including stroke services [35, 36]. The eight domains align closely with the core messages from the workshops, for example: sense making (built through trusting relationships); embodiment (feeling that staff see them as more than just a body); insiderness (engaging with patients as people, rather than just tasks to accomplish); uniqueness (taking an interest in what matters to their patient) and agency (supporting autonomy) [36]. Research investigating interventions that deliver person‐centred care, as opposed to more paternalistic, bio‐medical, task‐focused care, has found it is associated with improved patient satisfaction, improved staff well‐being and job satisfaction, and improved quality of care [33]. A theme from qualitative research into compassionate or humanised care interventions is that this way of working enables healthcare staff to reconnect with their values and aspirations as healthcare workers [37, 38].

Lived experience stakeholders felt strongly that supporting emotional recovery, or at least not worsening someone's psychological distress, was the role of the whole stroke team. This aligns with best practice guidelines that state that all clinical staff should have an awareness of psychological problems following a stroke and the skills necessary for providing ‘Level 1’ (i.e., non‐specialist) psychological support, such as active listening [13]. In fact, workshop members went further than these guidelines, suggesting that non‐clinical staff can also have an important role. For example, there is some evidence that hospital cleaners value relational moments and consider communication with patients as much a part of high‐quality work as a clean room, yet healthcare systems consider such moments as time‐inefficient and ‘out of protocol’ [39]. There is also some evidence that interactions with hospital receptionists can support or damage well‐being [38]. It may be that shifting the culture to value and include all staff, both clinical and non‐clinical, may be part of the way forward in promoting a humane culture within stroke care. The combination of aphasia and low mood appears to be a particularly challenging combination for staff [4]. It is encouraging that recent research suggests it is possible to adapt psychological therapies so that they are accessible for people with aphasia [14, 16, 40]. The current study adds to the body of evidence that suggests that people with aphasia are also likely to find psychological benefit when the stroke unit culture prioritises relational work, staff actively engage with people with aphasia, and it is valued and prioritised for staff to provide reassurance and empathy.

A limitation of the study is that although the researchers were speech and language therapists, we did not include other healthcare staff as part of the process [41]. Since the films were aimed at healthcare staff, involving them as key stakeholders in the co‐production process would likely have strengthened the acceptability and utility of the films in changing staff behaviour. A criticism levelled at co‐produced research is the lack of robust evaluation in terms of improving healthcare outcomes [19]. A logical next step in the collaborative research process evaluation would be evaluating whether the films can influence healthcare outcomes. A further limitation is that the workshops were not recorded: while the contemporaneous notes were detailed, they were not as comprehensive as a verbatim transcript, which may have introduced bias.

Within co‐produced research, creative approaches such as creating ‘personas’ or techniques such as ‘SWIM’ (someone who isn't me) are often used [19, 41]. These can create a useful distance when discussing personal topics, as well as inviting more perspectives into the room. Within the current project, facilitators had created possible personas to stimulate discussion. However, lived experience stakeholders preferred to speak frankly and openly about themselves, potentially facilitated by the clinical backgrounds of the facilitators. The facilitators made the decision to share their own healthcare experiences to a limited extent to equalise power relationships within the group; this was a notably different approach from their normal practice either as clinicians or researchers.

There is an argument that those involved in co‐produced research may be an unrepresentative minority, which potentially delegitimises it. Common barriers to inclusion from a wider patient body include travel (costs and access), geographical location and taking time off work [42]. To broaden inclusion, researchers have a role in ensuring that involvement opportunities are as accessible as possible [41]. Through conducting workshops online, we were able to include people from different geographical locations, and those who find public transport onerous. We timed workshops to be during lunch breaks to facilitate the two members who worked. Nonetheless, the decision to conduct workshops online meant we excluded those without digital access. While we included both men and women, younger and older, and white and ethnic minority members, we nonetheless acknowledge that members likely had higher levels of education than the UK stroke population, although we cannot know this as personal data was not collected. It has been suggested that we require a ‘strange mix of representativeness, diversity, ordinariness, knowledge and expertise’ (p46) from service user co‐workers [43]. We suggest there are specific skills and aptitudes, including empathy, reflection and a desire to see changes that will benefit others. As such, the qualities needed from service user co‐workers go beyond representation.

It is argued that there is philosophical legitimacy to co‐produced research and that ‘those affected by research are best placed to design and deliver it.’ [41] Through careful listening to different stakeholders' experiences, values and ideas, we believe the final output was stronger and richer than had we taken a more traditional ‘top down’ approach to research. There were challenges: the final outputs were different from those described in the original funding application, and as such, our original aim of informing specialist training through a lived experience perspective was not met. This is both a strength and a limitation of co‐created research, where solutions emerge in an iterative manner from the fluid process and the relationships built [41]. The non‐standard research paradigm also required the researcher stakeholders to reflect carefully on ethics and clinical duty of care, while also enabling equity of voice and roles within the process.

## 5 Conclusion

This co‐created study explored the views of people with aphasia and their family members on how best to influence healthcare staff so that they are more able to address the psychological needs of people following a stroke and aphasia; the co‐creative research process enabled the study to evolve in line with the priorities of people with aphasia leading to a series of co‐produced films. The lived experience stakeholders requested that the researchers share their messages ‘with the whole world’. As such, they entrusted the researchers to disseminate their messages as widely as possible to influence care.

## Author Contributions

**Sarah Northcott:** conceptualisation, investigation, funding acquisition, writing – original draft, writing – review and editing, formal analysis, project administration, supervision, methodology, resources. **Amanda Comer:** formal analysis, writing – review and editing, investigation, resources. **Abi Roper:** conceptualisation, methodology, writing – review and editing, data curation. **Lydia Davis:** conceptualisation, investigation, writing – review and editing. **Katerina Hilari:** conceptualisation, writing – review and editing, methodology, supervision.

## Conflicts of Interest

The authors declare no conflicts of interest.

## Data Availability

The authors have nothing to report.
